# Transcription factor glioma-associated oncogene homolog 1 is required for transforming growth factor-β1-induced epithelial-mesenchymal transition of non-small cell lung cancer cells

**DOI:** 10.3892/mmr.2015.3150

**Published:** 2015-01-07

**Authors:** HUA LI, LI-JUN DA, WEI-DONG FAN, XIAO-HONG LONG, XIAN-QUAN ZHANG

**Affiliations:** Department of Oncology, The Second Affiliated Hospital, Chongqing Medical University, Chongqing 400010, P.R. China

**Keywords:** non-small cell lung cancer, transforming growth factor-β1, human glioma-associated oncogene homolog 1 transcription factor, epithelial-mesenchymal transition

## Abstract

Epithelial-mesenchymal transition (EMT) is the process by which epithelial cells depolarize and acquire a mesenchymal phenotype, and is a common early step in the process of metastasis. Patients with lung cancer frequently already have distant metastases when they are diagnosed, highlighting the requirement for early and effective interventions to control metastatic disease. Transforming growth factor-β1 (TGF-β1) is able to induce EMT, however the molecular mechanism of this remains unclear. In the current study, TGF-β1 was reported to induce EMT and promote the migration of non-small cell lung cancer (NSCLC) cells. A notable observation was that EMT induction was accompanied by the upregulation of human glioma-associated oncogene homolog 1 (Gli1) mRNA and protein levels. Furthermore, Gli1 levels were depleted by small interfering RNA, and the Gli1 inhibitor GANT 61 attenuated the TGF-β1-mediated induction of EMT and cell migration. The results of the current study suggest that Gli1 regulates TGF-β1-induced EMT, which may provide a novel therapeutic target to inhibit metastasis in patients with NSCLC.

## Introduction

Lung cancer is the leading cause of cancer-associated mortality worldwide (1) and ~85% of cases of lung cancer are classified as non-small cell lung cancer (NSCLC) (2). Despite improvements in diagnostic and therapeutic strategies, the prognosis for patients with NSCLC remains poor, with a 5-year survival rate of 8–14% (3). The primary cause of lung cancer-associated mortality is metastasis, and the majority of patients with NSCLC have begun to develop metastatic disease by the time they are diagnosed (1,2). Thus, effective NSCLC therapies must include strategies to control metastatic disease. Such strategies may be improved by a more thorough understanding of the underlying mechanisms of NSCLC metastasis.

Epithelial-mesenchymal transition (EMT) is an early event in the metastatic progression of a number of types of epithelial cancer, such as lung cancer (4–10). EMT is the process by which epithelial cells transition from a typical epithelial phenotype (polarized and adherent) to a mesenchymal phenotype (spindle-shaped and motile). EMT results in clear alterations in the morphology, adhesive properties and gene expression of cells, including the upregulation of vimentin, N-cadherin and fibronectin, in addition to the downregulation of E-cadherin and cytokeratin (4,5). Additionally, the mesenchymal state during EMT is associated with a higher capacity for migration and invasion (11).

The process of EMT is regulated by a complex system of signal transduction pathways. One key regulator of EMT in lung cancer is the transforming growth factor-β (TGF-β) signaling pathway (11,12). In addition to TGF-β, the Hedgehog (Hh) signaling pathway is known to participate in EMT, however the precise role of this pathway in EMT remains unclear (5). The Hh signaling pathway has been reported to be activated in a number of human tumors, including NSCLC and metastatic disease (13) and ultimately activates the transcription factor human glioma-associated oncogene homolog 1 (Gli1). Gli1 is also activated by other cancer-associated signaling pathways, such as the receptor tyrosine kinase and phosphoinositide 3-kinase (PI3K) pathways (14).

Despite its association with Hh signaling, the specific function of Gli1 in EMT remains to be fully elucidated. In the current study, the role of Gli1 in TGF-β-induced EMT was investigated in NSCLC cell lines. Gli1 levels in NSCLC cells that underwent TGF-β1-induced EMT were measured, and the effect of small interfering RNA (siRNA)- or pharmacological agent-mediated inhibition of Gli1 activity on TGF-β1-induced EMT was analyzed. To investigate this, alterations in morphology, phenotypic markers, invasion and migratory capability were measured.

## Materials and methods

### Cell lines and reagents

The lung cancer cell lines A549, H460 and SK-MES-1 were purchased from the American Type Culture Collection (Manassas, VA, USA) and cultured in RPMI-1640 medium containing 10% fetal bovine serum (FBS) (Gibco Life Technologies, Carlsbad, CA, USA) at 37°C in a humidified atmosphere with 5% CO_2_. Recombinant human TGF-β1 and GANT 61 were purchased from PeproTech, Inc. (Rocky Hill, NJ, USA). Phase contrast images of A549 cells were acquired using an inverted phase contrast microscope (IX53; Olympus Corporation, Tokyo, Japan) subsequent to incubation of the cells with 0, 1, 5 or 10 ng/ml TGF-β1 for 48 h. For western blot and immunofluorescent analysis, polyclonal rabbit anti-human Gli1 (ab49314), polyclonal rabbit anti-human E-cadherin (ab15148), monoclonal rabbit anti-human vimentin (ab16700), polyclonal rabbit anti-human β-actin (ab1801) and horseradish peroxidase (HRP)-conjugated anti-rabbit secondary antibodies were purchased from Abcam (Cambridge, MA, USA).

### siRNA transfection and drug treatments

GFP-siRNA specific for Gli1 and nonspecific GFP-siRNA were diluted in diethylpyrocarbonate (DEPC)-treated water (all from Life Technologies, Grand Island, NY, USA). The siRNA was used to deplete Gli1 mRNA and protein levels in the A549 cells. A549 cells were transfected with DEPC-treated water (control group), a nonspecific control siRNA (si-VE group) or Gli1-specific siRNA (si-Gli1 group). A549 cells were cultured until 60–70% confluence was reached and were then transfected with siRNA using X-tremeGENE siRNA (Roche Diagnostics, Basel, Switzerland) and OptiMEM medium (Life Technologies) according to the manufacturer’s instructions. Following 6-h transfection, the cells were incubated in RPMI-1640 with 10% FBS and 5 ng/ml TGF-β1. At 24 h subsequent to transfection, the cells were imaged using the inverted phase contrast microscope to assess cell morphology, while transfection efficiency was assessed by detecting green fluorescent protein (GFP) fluorescence using a confocal laser-scanning microscope (OLS3100; Olympus, Corporation). Following a 48-h resting period, cells were collected for Transwell invasion assays, reverse transcription-quantitative polymerase chain reaction (RT-qPCR) and western blot analyses. For treatment with GANT 61, the H460 and SK-MES-1 cells were incubated with 10% FBS for 24 h and stimulated with 5 ng/ml TGF-β1 for 48 h in the presence or absence of 10 μM GANT 61.

### RNA isolation and RT-qPCR

Total RNA from NSCLC cells was isolated using RNAiso Plus extraction reagent and cDNA was produced using PrimeScript Reverse Transcriptase (Takara Bio, Inc., Otsu, Japan) according to the manufacturer’s instructions. RT-qPCR reactions were then conducted with SYBR Premix Ex Taq II (Takara Bio, Inc.) in 25 μl reactions with 2 μl cDNA and 0.4 μM each of the forward and reverse primers using the following thermocycling conditions: 95°C for 30 sec, 95°C for 5 sec and 60°C for 30 sec. Data was collected over 40 cycles and all expression levels were normalized to β-actin. The following primers were used: Gli1, F 5′-CTG GAC CTG CAG ACG GTT ATC-3′ and R 5′-AGC CTC CTG GAG ATG TGC AT-3′; β-actin, F 5′-TGA CGT GGA CAT CCG CAA AG-3′ and R 5′-CTG GAA GGT GGA CAG CGA GG-3′ (Sangon Biotech Co., Ltd., Shanghai, China).

### Western blot analysis

For biochemical analysis, cells were washed with ice-cold phosphate-buffered saline (PBS; Beyotime Institute of Biotechnology, Jiangsu, China) and lysed in radioimmunoprecipitation assay lysis buffer [50mM Tris, pH 7.4; 150mM NaCl; 1% Triton X-100; 1% sodium deoxycholate; 0.1% sodium dodecyl sulfate (SDS); Beyotime Institute of Biotechnology]. The lysates were kept on ice for 30 min and clarified by centrifugation at 12,000 × g for 25 min at 4°C. The clarified lysate was then collected and the vimentin, β-actin, E-cadherin and Gli1 proteins were separated by 12% SDS-polyacrylamide gel electrophoresis (30–100 μg protein/lane) and transferred to a polyvinylidine fluoride membrane (all from Beyotime Institute of Biotechnology). Subsequent to transfer, the membranes were incubated in 5% milk for 1 h, and then with the Gli1 (2.5 μg/ml), E-cadherin (1:500), vimentin (1:500) or β-actin (1:1000) antibodies diluted in non-fat milk. The membranes were then washed with Tris-buffered saline with Tween-20 (Beyotime Institute of Biotechnology) and incubated with the HRP-conjugated secondary antibodies. Immunoreactive proteins were visualized using a BeyoECL Plus kit (Beyotime Institute of Biotechnology).

### Immunofluorescence

For the immunofluorescence analysis, NSCLC cells were seeded onto glass slides 24 h prior to treatment and were transfected with TGF-β1 and siRNA. Following treatment for 24 h, cells were fixed with 4% paraformaldehyde (Beyotime Institute of Biotechnology) for 15 min, washed with PBS, and permeabilized with 0.1% Triton X-100 (Beyotime Institute of Biotechnology) for 30 sec. To examine cell-surface expression of E-cadherin, the cells were not permeabilized following fixation. Cells were incubated with 5% sheep serum albumin (Beyotime Institute of Biotechnology) for 1 h to block non-specific proteins. Subsequent to blocking, cells were incubated with the Gli1, E-cadherin, vimentin or β-actin antibodies, washed with PBS three times, and then incubated with fluorescein isothiocyanate-conjugated goat anti-rabbit and tetramethylrhodamine isothiocyanate-conjugated goat anti-rabbit secondary antibodies (ZF-0311; Zhongshan Golden Bridge Biotechnology Co., Ltd., Beijing, China) diluted in distilled water for 1 h at 37°C. The stained cells were placed on a coverslip in mounting medium (Beyotime Institute of Biotechnology) with 4′,6-diamidino-2-phenylindole (Beyotime Institute of Biotechnology) to label the nuclei. Cells were imaged using the OLS3100 fluorescence microscope (magnification, ×400).

### Transwell cell migration and invasion assay

The invasive and migratory ability of NSCLC cells was assessed using Matrigel-coated or uncoated chambers (Corning Incorporated, New York, NY, USA). At 48 h subsequent to transfection or GANT 61 (PeproTech, Inc.) treatment, 1×10^5^ cells were seeded into the upper Transwell chamber, while the lower chamber was filled with RPMI-1640 supplemented with 20% FBS and 5 ng/ml TGF-β1. The chambers were incubated at 37°C with 5% CO_2_ for 24 h. Cells on the upper surface of the filter were removed using a cotton swab, while cells that invaded/migrated to the lower surface of the filter were fixed with 4% paraformaldehyde for 15 min at room temperature, and stained with 0.1% crystal violet (Beyotime Institute of Biotechnology) for 30 min. Crystal violet-stained cells were observed and images were captured under the phase contrast microscope, and counted in five randomly selected fields (magnification, ×100). The experiments were performed in triplicate and each experiment was performed three times.

### Wound healing cell migration assay

NSCLC cells were seeded into six-well plates and allowed to form confluent monolayers. The cell monolayers were scratched using a 200 μl pipette tip to create a wound, washed three times with PBS and incubated in RPMI-1640 supplemented with 0, 1, 5 and 10 ng/ml TGF-β1. Wound width was measured using phase contrast microscopy and images were captured immediately subsequent to the addition of TGF-β1 (0 h) and at 48 h of incubation.

### Statistical analysis

Statistical analysis was conducted using SPSS, version 18.0 (SPSS, Inc., Chicago, IL, USA). All results are presented as the mean ± standard deviation. Statistical analysis was performed using analysis of variance with the Tukey-Kramer post-hoc test. P<0.05 was considered to indicate a statistically significant difference.

## Results

### TGF-β1 induces EMT in NSCLC cells

**Figure 1:**
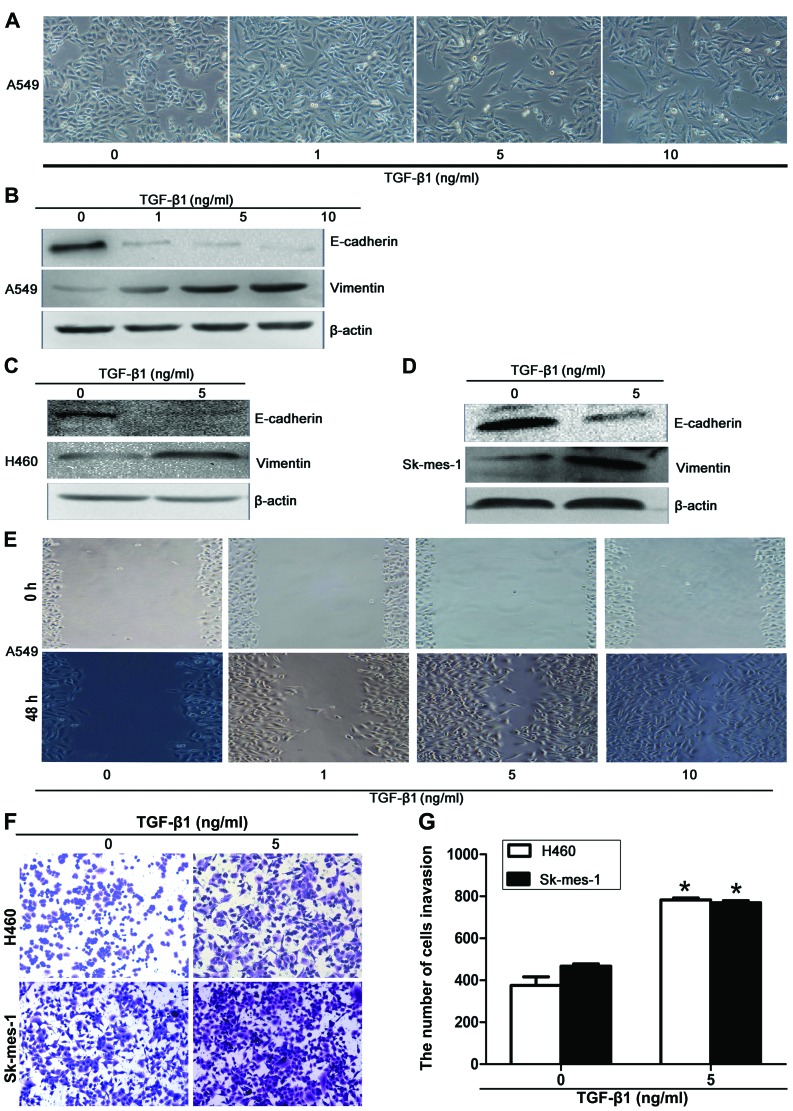
TGF-β1-induced EMT in the A549 cell line. (A) Phenotypic alterations induced by treatment with 0, 1, 5 or 10 ng/ml TGF-β1 for 48 h (magnification, ×100). Western blot analysis to determine the protein levels of E-cadherin, vimentin and β-actin in (B) A549, (C) H460 and (D) SK-MES-1 cells treated with TGF-β1 for 48 h. (E) The migratory capacity of A549 cells was assessed by a wound healing assay. Phase contrast microscope images captured at 0 and 48 h subsequent to TGF-β1 treatment; magnification, ×200. Transwell cell invasion assay to analyze the invasive capacity of H460 and SK-MES-1 cells treated with 5 ng/ml TGF-β1 for 48 h. Invasive cells were (F) imaged with crystal violet and (G) quantified. ^*^P<0.05 vs. control. TGF-β1, transforming growth factor-β1; EMT, epithelial-mesenchymal transition.

Epithelial cancer cells undergo EMT early in the process of metastasis (15). A549 cells undergo EMT in response to TGF-β1 exposure (16,17), hence these cells can be used as a model to study EMT in NSCLC. To establish the role of TGF-β1 in EMT, A549 cells were treated with 0, 1, 5 or 10 ng/ml TGF-β1 for 48 h and the morphological alterations occurring were assessed by phase contrast microscopy. The untreated A549 cells were observed to exhibit classic epithelial morphology, whereas subsequent to 48-h TGF-β1 treatment, cell morphology was clearly altered. The cells acquired a mesenchymal phenotype, becoming elongated, spindle-shaped cells with reduced cell-cell contacts (Fig. 1A). The extent of these morphological alterations was dose-dependent for TGF-β1, suggesting that the effects observed were a result of TGF-β1 treatment.

The phenotypic alterations occuring suggested that A549 cells had undergone EMT, thus the expression of specific EMT markers, including the epithelial marker E-cadherin and the mesenchymal marker vimentin were measured (7,11). Following the treatment of A549 cells with a range of concentrations of TGF-β1 for 48 h, E-cadherin and vimentin expression was assessed by western blot analysis. TGF-β1 treatment led to reduced expression of the E-cadherin and increased expression of the vimentin compared with the control, in a concentration-dependent manner (Fig. 1B). Similar results were obtained in the other NSCLC cell lines, with 5 ng/ml TGF-β1-treatment of H460 and SK-MES-1 cells for 48 h resulting in increased expression levels of vimentin but a reduction in E-cadherin expression, compared with untreated cells (Fig. 1C and D). These data suggest that TGF-β1 induces EMT in NSCLC cells.

The development of metastatic disease is the most frequent cause of cancer-associated mortality (18). EMT is often associated with metastasis, as the invasive and migratory capacity of cells increases following the induction of EMT. To analyze the migratory capacity of A549 cells following TGF-β1 treatment, wound healing assays were conducted. In this assay, it was observed that TGF-β1 treatment led to increased migration after 48 h compared with untreated cells (Fig. 1E). To assess the effect of TGF-β1 on the invasive capacity of NSCLC cells, Transwell invasion assays were conducted using TGF-β1-treated H460 and SK-MES-1 cells, which exhibited increased invasion compared with the control (Fig. 1F and G). These results indicate that TGF-β1 induces enhanced migration and invasion of NSCLC cells, which is consistent with the observed phenotypic alterations. These data support an involvement of TGF-β1 in the induction of EMT in NSCLC cells.

### Upregulation of Gli1 expression levels in TGF-β1-stimulated NSCLC cells

**Figure 2:**
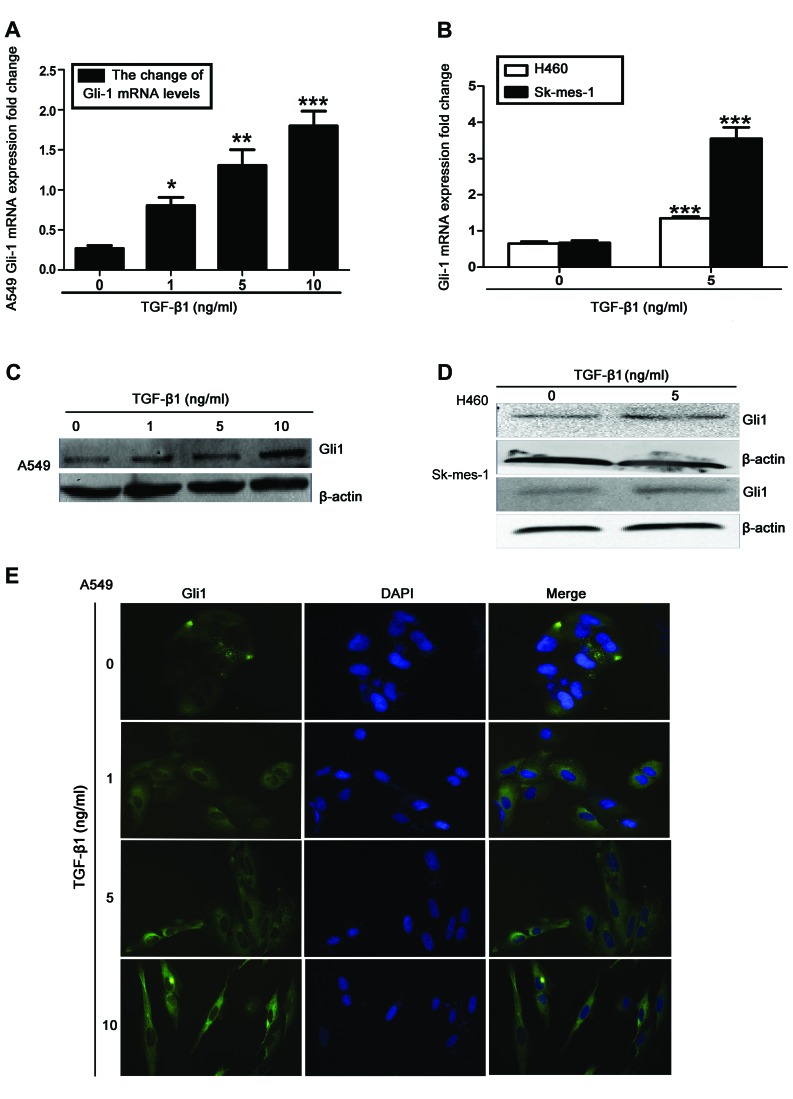
TGF-β1-treated NSCLC cells demonstrated an upregulation of Gli1 mRNA and protein levels. Gli1 mRNA levels assessed by RT-qPCR in (A) A549 and (B) H460 and SK-MES-1 cells treated for 48 h with TGF-β1. All values are presented as the mean, error bars represent the standard deviation, ^*^P<0.05, ^**^P<0.005 and ^***^P<0.001 vs. control. Gli1 protein levels in (C) A549 and (D) H460 and SK-MES-1 cells stimulated with TGF-β1 were examined using western blotting, with β-actin as a loading control. (E) Immunofluorescence staining (magnification, ×400). All experiments were performed 3 times. TGF-β1, transforming growth factor-β1; NSCLC, non-small cell lung cancer; Gli1, glioma-associated oncogene homolog 1; RT-qPCR, reverse transcription-quantitative polymerase chain reaction.

Gli1 is commonly observed to be overexpressed in NSCLC, and has been implicated in the induction of EMT and metastasis (13,19–21). Therefore, the role of Gli1 was investigated in TGF-β1-induced EMT. Using RT-qPCR and western blot analysis, it was observed that TGF-β1 stimulation significantly increased Gli1 mRNA and protein levels in A549, H460 and SK-MES-1 cells, compared with untreated cells (Fig. 2A–D). A similar induction of Gli1 was observed in the immunofluorescence analysis of TGF-β1-treated A549 cells (Fig. 2E). These results indicate that Gli1 upregulation occurs during TGF-β1-induced EMT in NSCLC cells.

### Effect of Gli1-siRNA and GANT 61 treatment on Gli1 induction by TGF-β1 in NSCLC cells

**Figure 3:**
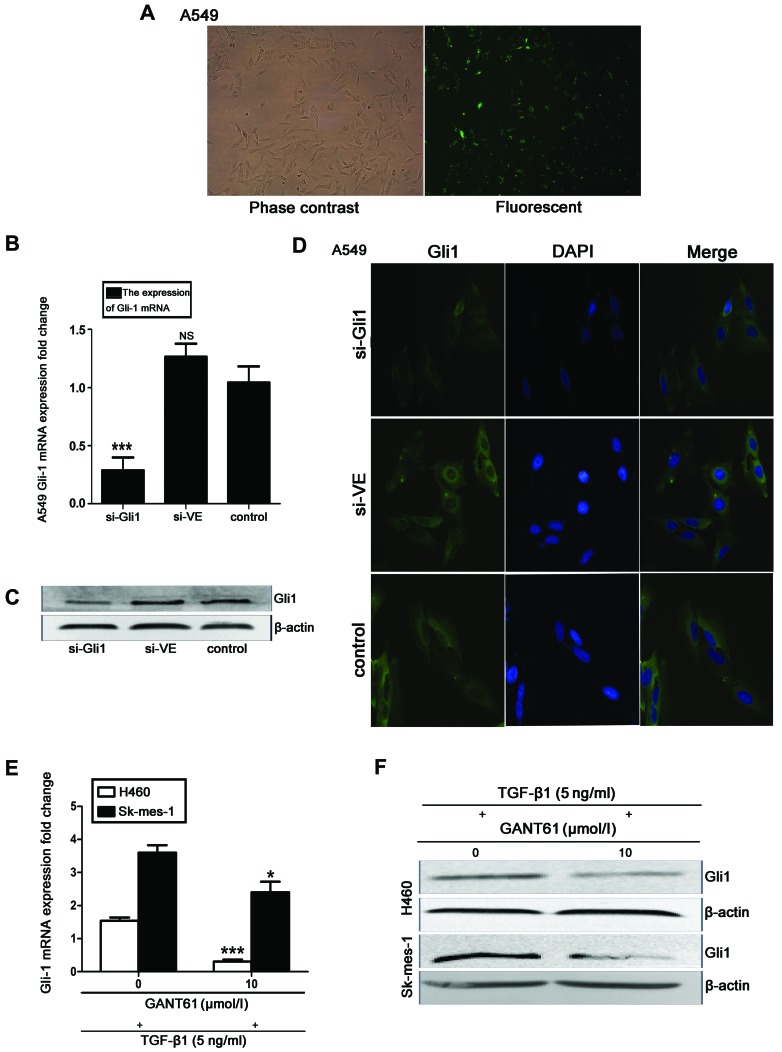
Effect of Gli1-siRNA and GANT 61 on Gli1 expression in TGF-β1-induced NSCLC cells. (A) Confocal laser scanning microscope images to assess transfection efficiency. (B) Gli1 mRNA expression levels were assessed by RT-qPCR. (C) Western blot analysis of Gli1 protein expression in the si-Gli1, si-VE and control groups, with β-actin as the loading control. (D) Immunofluorescence staining (magnification, ×400). All experiments were performed 3 times. (E) RT-qPCR assessment and (F) western blot analysis of H460 and SK-MES-1 cells treated with the Gli1 inhibitor GANT 61 (10 μM) for 48 h, followed by 48 h stimulation with TGF-β1. All values are presented as the mean, error bars represent the standard deviation; NS, not significant, ^*^P<0.05, ^**^P<0.005 and ^***^P<0.001 vs. control. Gli1, glioma-associated oncogene homolog 1; si-Gli1, Gli1 siRNA group; si-VE, nonspecific siRNA group; TGF-β1, transforming growth factor-β1; NSCLC, non-small cell lung cancer; RT-qPCR, reverse transcription-quantitative polymerase chain reaction.

To determine the function of Gli1 in TGF-β1-induced EMT, siRNA was used to deplete Gli1 mRNA and protein levels in A549 cells. The cells were transfected with DEPC-treated water (control), Gli1-specific siRNA (si-Gli) or nonspecific control siRNA (si-VE) for 24 h. Fluorescence microscopy analysis indicated that the siRNAs were efficiently transfected, as demonstrated by the presence of GFP expression (Fig. 3A). Subsequent to a 48-h resting period, Gli1 mRNA expression levels were significantly suppressed by Gli1-specific siRNA compared with control, as measured by RT-qPCR (P<0.001; Fig. 3B). This siRNA-mediated depletion of Gli1 was further confirmed by western blotting (Fig. 3C) and immunofluorescence analysis (Fig. 3D). As an alternative to siRNA-mediated depletion of Gli1, the effects of pharmacological inhibition of Gli1 were investigated using the Gli1-specific inhibitor GANT 61 in TGF-β1-treated H460 and SK-MES-1 cells. Cells were treated with 10 μM GANT 61 for 48 h prior to stimulation with 5 ng/ml TGF-β1 for an additional 48 h. This was demonstrated by RT-qPCR and western blot analysis to downregulate Gli1 mRNA and protein levels in the H460 (P<0.001) and SK-MES-1 (P<0.05) cell lines, compared with controls (Fig. 3E and F).

### Effects of Gli1 inhibition on TGF-β1-induced EMT of NSCLC cells

**Figure 4:**
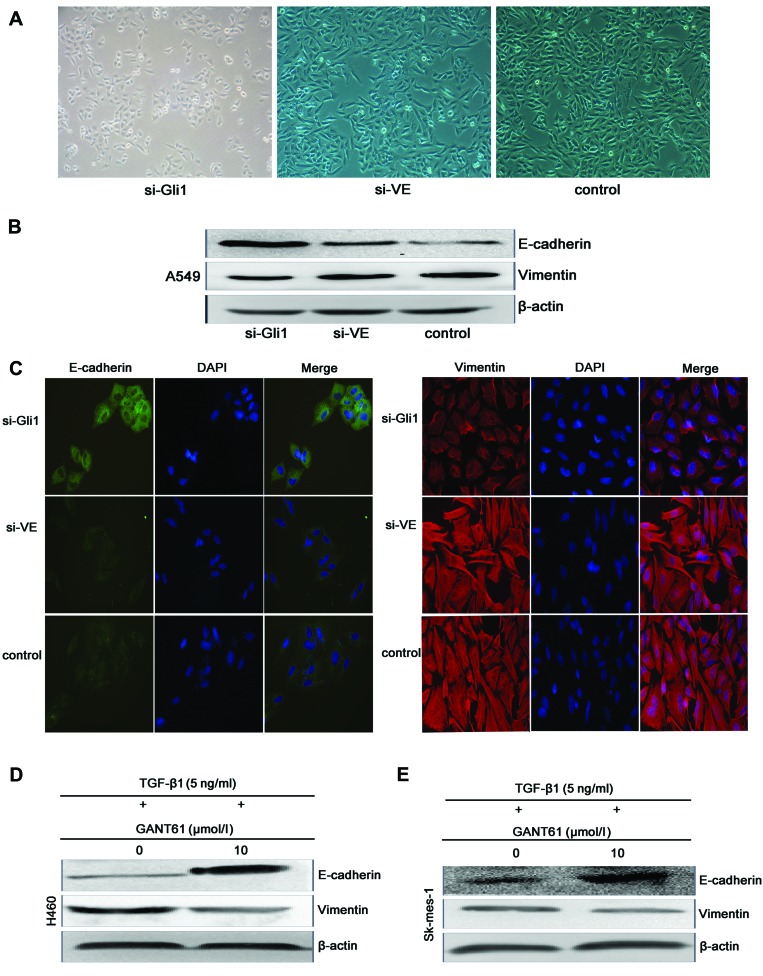
Effects of Gli1 on TGF-β1-induced EMT of NSCLC cells. (A) Phase contrast microscopy used to assess the morphology of A549 cells 24 h subsequent to si-Gli1, si-VE or blank transfection 18 h following TGF-β1-stimulation. (B) Western blot analysis and (C) immunofluorescence staining of E-cadherin, vimentin and β-actin levels 48 h subsequent to transfection. The immunofluorescence experiments were performed 3 times and images represent a magnification of ×400. Western blot analysis to assess E-cadherin and vimentin expression in (D) H460 and (E) SK-MES-1 cells treated with the Gli1 inhibitor GANT 61 (10 μM) for 48 h and stimulated with 5 ng/ml TGF-β1 for 48 h. Gli1, glioma-associated oncogene homolog 1; TGF-β1, transforming growth factor-β1; EMT, epithelial-mesenchymal transition; NSCLC, non-small cell lung cancer; si-Gli1, Gli1 siRNA group; si-VE, nonspecific siRNA group.

To determine if Gli1 participated in the induction of EMT in NSCLC cells, phase contrast microscopy was used to assess the morphology of A549 cells 24 h subsequent to si-Gli1, si-VE or blank transfection, and 18 h subsequent to TGF-β1 stimulation (Fig. 4A). The expression of EMT markers in TGF-β1 following siRNA-mediated depletion of Gli1 was also assessed. Western blot and immunofluorescence analysis demonstrated that, compared with cells transfected with control siRNA, E-cadherin expression was elevated in cells transfected with Gli1 siRNA, while vimentin protein levels were reduced (Fig. 4B and C). The effects of pharmacological inhibition of Gli1 on TGF-β1-induced EMT in H460 and SK-MES-1 cells were also investigated. An elevation of E-cadherin protein levels and a reduction of vimentin expression was observed following 48-h GANT 61 treatment in H460 and SK-MES-1 cells, compared with untreated cells (Fig. 4D and E). These results suggest that the downregulation of Gli1 inhibits TGF-β1-induced EMT of NSCLC cells.

### Downregulation of Gli1 reduces the invasive and migratory capacity of the TGF-β1-stimulated NSCLC cells

**Figure 5:**
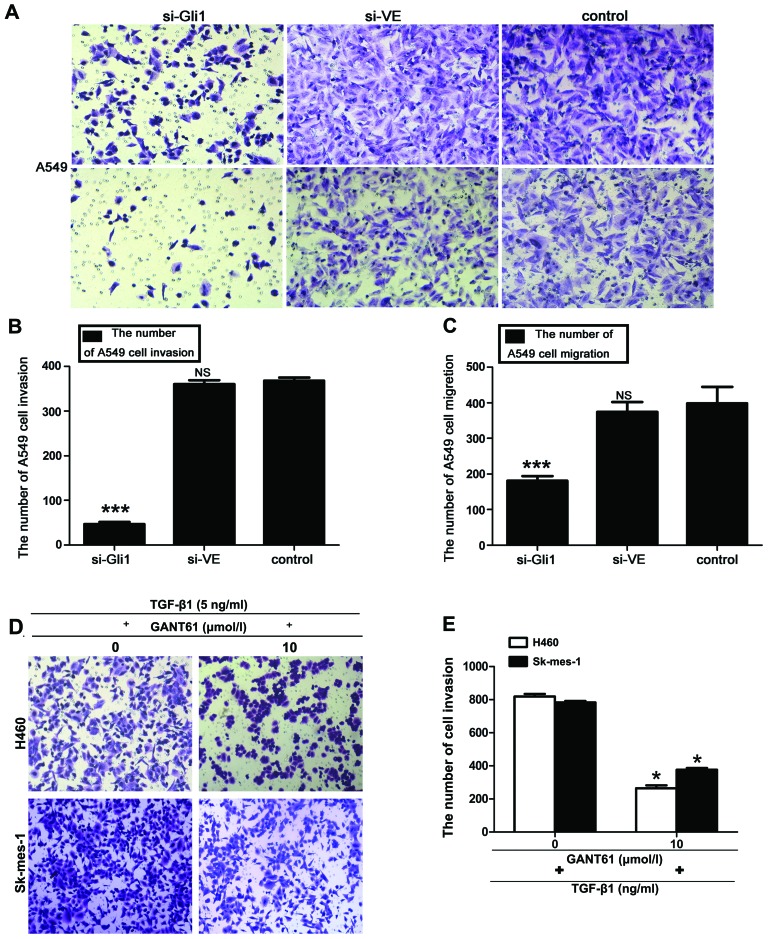
Inhibition of Gli1 reduced the invasive and migratory capacity of TGF-β1-induced NSCLC cells. (A) Cells were seeded 48 h subsequent to si-Gli1, si-VE, or blank transfection into the upper transwell chambers, which were either pre-coated with 20 mg Matrigel (invasion assay), or uncoated (migration assay). Following a 24-h resting period, cells which had migrated (upper panels) or invaded (lower panels) were stained with crystal violet and imaged with a phase contrast microscope (magnification, ×100). Five fields were randomly chosen from each filter, and values representing the mean numbers of cells which had (B) invaded or (C) migrated were obtained. Each experiment was performed in triplicate. (D and E) Transwell Matrigel invasion assay results from TGF-β1-stimulated H460 and SK-MES-1 cells treated with 10 μM GANT 61. All values represent the mean number of cells, error bars represent the standard deviation. NS, not significant; ^*^P<0.05, ^**^P<0.005 and ^***^P<0.001 vs. control. Gli1, glioma-associated oncogene homolog 1; TGF-β1, transforming growth factor-β1; NSCLC, non-small cell lung cancer; si-Gli1, Gli1 siRNA group; si-VE, nonspecific siRNA group.

Given the effects of Gli1 inhibition on the expression of EMT markers, the effect of Gli1 siRNA on the invasive and migratory abilities of TGF-β1-induced A549 cells was investigated. Compared with cells transfected with non-specific control siRNA and untransfected cells, cells with Gli1 siRNA exhibited significantly reduced invasion and migration in a Transwell assay (P<0.001; Fig. 5A–C), suggesting that Gli1 is required for migration in response to TGF-β1. The effects of Gli1 inhibition on the invasive capacity of H460 and SK-MES-1 cells were also investigated. Suppression of Gli1 using GANT 61 significantly inhibited the ability of H460 and SK-MES-1 cells to invade through Matrigel compared with untreated cells (P<0.05; Fig. 5D and E). These data suggest that Gli1 contributes to the enhanced migration and invasion of TGF-β1-stimulated NSCLC cells.

## Discussion

EMT is an important biological process that allows cancer cells to develop metastatic characteristics. One of the major alterations that occurs during EMT is the loss of the cell-cell adhesion molecule E-cadherin and overexpression of vimentin, N-cadherin and MMP-9 (6). In the current study, A549 NSCLC cells were demonstrated to undergo morphological alterations associated with EMT, following stimulation with TGF-β1, in a dose-dependent manner. Following 48-h TGF-β1 stimulation, the A549, H460 and SK-MES-1 cells were observed to exhibit a mesenchymal phenotype, with elongated, spindle-shaped cells and a reduction in cell-cell contacts. These morphological alterations were accompanied by the downregulation of E-cadherin and upregulation of vimentin, which is consistent with classical EMT.

In addition to these morphological alterations, EMT is associated with increased motility and invasive ability. Consistent with this, the current study observed that TGF-β1 treatment led to an increase in the migratory behavior of A549 cells compared with untreated cells in a wound healing assay. Furthermore, TGF-β1 enhanced the invasive ability of H460 and SK-MES-1 cells in a Transwell Matrigel invasion assay. Taken together, these data indicate that TGF-β1 promotes EMT and its associated metastatic behavior in NSCLC cells.

To investigate the molecular mechanism of TGF-β1-induced EMT in NSCLC, the current study focused on Gli1. Notably, TGF-β1 stimulation was observed to upregulate Gli1 expression in A549 cells in a dose-dependent manner, as assessed by western blotting, RT-qPCR and immunofluorescence analysis, suggesting that Gli1 may serve an important function in TGF-β1-induced EMT. Inhibition of Gli1 using siRNA or the Gli1 inhibitor GANT 61 prevented morphological alterations in NSCLC cells following TGF-β1 stimulation. Furthermore, inhibition of Gli1 attenuated the induction of the mesenchymal marker vimentin, and upregulated the epithelial marker E-cadherin following TGF-β1 treatment. In addition, inhibition of Gli1 reduced the migratory and invasive capacity of NSCLC cells. These results suggest that Gli1 is important in the induction of EMT by TGF-β1 and that inhibition of Gli1 may reverse the EMT phenotype in NSCLC cells, thus potentially reducing metastasis.

TGF-β1 is a multifunctional cytokine that is closely associated with cell growth, fibrosis and tumorigenesis. It is also able to promote EMT and tumor cell metastasis (11,22), and this has been demonstrated in several cancer cell lines (23–26). The Gli1 protein is a zinc finger transcription factor that is activated by the Hh signaling pathway, which involves Hh proteins, the Patched protein, the Smoothened (Smo) protein and the five-zinc finger Gli transcription factor family (Gli1, Gli2 and Gli3) (27,28). Gli1 and Gli2 act as the main activators of Hh-target genes, while Gli3 acts as a repressor of Hh target genes (29,30). The Hh signaling pathway is implicated in developmental processes and tumor malignancies (31,32,34), and the Gli family of transcription factors mediate a number of important cellular processes, including EMT, migration, metastasis and tumorigenesis (21). In NSCLC specifically, Gli1 has been demonstrated to be elevated in tumor tissue samples and NSCLC cells lines (13,33). In addition, upregulation of Hh signaling has been indicated to contribute to TGF-β1-induced EMT in NSCLC cells (19). These data are consistent with the observations of the current study, demonstrating that Gli1 levels are elevated in TGF-β1-stimulated NSCLC cells that have undergone EMT, thus it is suggested that Gli1 is important in EMT downstream of TGF-β1.

The current study suggests that Hh signaling, and Gli1 in particular, may provide a novel therapeutic target in NSCLC. As the Gli1 transcription factor mediates the terminal effects of the Hh pathway, the upstream positive regulator of Gli1 (Smo) has been investigated as a therapeutic target, with several Smo-targeted small molecule inhibitors currently undergoing clinical trials. One Smo-targeted small molecule inhibitor, vismodegib (GDC-0449), has been demonstrated in phase I clinical trials to be suitable for the treatment of multiple types of cancer (35). In addition to Smo, other signaling pathways, such as oncogenic epidermal growth factor receptor-RAS-protein kinase B (AKT) signaling have been demonstrated to activate Gli1 (36). In particular, the PI3K/AKT and mitogen-activated protein kinase/extracellular-signal-regulated kinase (ERK)1/2 signaling pathways also regulate TGF-β1-induced EMT in A549 cells (37). However, the precise role Gli1 in PI3K and ERK signaling remains to be fully elucidated.

In conclusion, the results of the current study indicate that the loss of Gli1 in NSCLC cells dramatically attenuates TGF-β1-induced EMT. Gli1 appears to be required for the phenotypic alterations, in addition to the enhancement of motility and invasion associated with TGF-β1-induced EMT. The results of the present study suggest that inhibition of Gli1 may serve as a useful strategy to target metastatic disease in patients with NSCLC. Additionally, as EMT affects the sensitivity of NSCLC cell lines to common therapeutic agents including erlotinib, cisplatin and paclitaxel (38–40), targeting Gli1 may improve the efficacy of these therapies. EMT also affects the response to radiotherapy, including promoting radiation-induced fibrosis and post-radiotherapy-associated metastasis (40), which is suggested to occur through the loss of E-cadherin (41,42). Therefore, reversal of EMT via Gli1 inhibition may potentially resensitize NSCLC cells to chemotherapy and radiation, which would contribute to improved prognosis for patients.
